# Gender-based differences in the representation and experiences of academic leaders in medicine and dentistry: a mixed method study from Pakistan

**DOI:** 10.1186/s12909-024-05811-6

**Published:** 2024-08-16

**Authors:** Muhammad Shahzad, Brekhna Jamil, Mehboob Bushra, Usman Mahboob, Fayig Elmigdadi

**Affiliations:** 1 Faculty of Dentistry, Zarqa University, Zarqa, Jordan; 2 Institute of Basic Medical Sciences, Khyber Medical University, Peshawar, Pakistan; 3 Institute of Health Professions Education and Research, Khyber Medical University, Peshawar, Pakistan; 4 Department of Oral and Maxillofacial Surgery, Peshawar Dental College, Peshawar, Pakistan

**Keywords:** Women, Leaders, Qualitative study, Pakistan

## Abstract

**Background:**

Research evidence suggests gender-based differences in the extent and experiences of academic leaders across the globe even in developed countries like USA, UK, and Canada. The under-representation is particularly common in higher education organizations, including medical and dental schools. The current study aimed to investigate gender-based distribution and explore leaders’ experiences in the medical and dental institutes in a developing country, Pakistan.

**Methods:**

A mixed-method approach was used. Gender-based distribution data of academic leaders in 28 colleges including 18 medical and 10 dental colleges of Khyber Pakhtunkhwa, Pakistan were collected. Qualitative data regarding the experiences of academic leaders (*n* = 10) was collected through semi-structured interviews followed by transcription and thematic analysis using standard procedures.

**Results:**

Gender-based disparities exist across all institutes with the greatest differences among the top-rank leadership level (principals/deans) where 84.5% of the positions were occupied by males. The gender gap was relatively narrow at mid-level leadership positions reaching up to as high as > 40% of female leaders in medical/dental education. The qualitative analysis found gender-based differences in the experiences under four themes: leadership attributes, leadership journey, challenges, and support.

**Conclusions:**

The study showed that women are not only significantly under-represented in leadership positions in medical and dental colleges in Pakistan, they also face gender-based discrimination and struggling to maintain a decent work life balance. These findings are critical and can have important implications for government, organizations, human resource managers, and policymakers in terms of enacting laws, proposing regulations, and establishing support mechanisms to improve gender-based balance and help current and aspiring leaders in their leadership journey.

**Supplementary Information:**

The online version contains supplementary material available at 10.1186/s12909-024-05811-6.

## Background

An inclusive and diverse workforce is crucial for organizational success in today’s modern world. However, despite a gradual increase in the representation of women in the workforce globally, gender discrimination and unequal gender ratios still exist in workplaces both horizontally (i.e., across different sectors and industries) and vertically (women are usually excluded from the top positions at the organizational level [1–3]. Workplace gender descrimination, in any farm, is not only illegal and against the basic human rights [4], it also contributes towards the lower socioeconomic status of women globally [5]. The under-representation of women, especially at leadership position is particularly common in higher education organizations, including medical and dental schools, as reported previously [6, 7]. Even those who reach a position in academic healthcare institutes publish research less frequently [8], receive fewer research grants [9], and have 23% fewer chances of promotion [10] than their male counterparts.

Recently, researchers tried to explore the extent of gender-based parity in leadership positions in academic medicine and sciences in many different countries of the world. Surprisingly, gender imbalance exists at all levels and nations, including countries ranking high on gender equality indices, such as Nordic countries [11]. According to a report by the European Commission, only 25% of the professors in European member states are women [12]. Similarly, in medical schools in the United States of America (USA), only 18% of the department heads are females [13]. Research studies also revealed that gender-based inequalities are multifaceted, and many extrinsic and intrinsic factors contributing to the issue have been identified [10, 14]. However, most research studies exploring women and leadership in academic medicine have been conducted in developed Western countries, including Europe, the USA, and Canada [15]. There is a dire need for research to document the extent and how male and female professionals develop as leaders during their careers in academic medicine and dentistry, especially from a non-western perspective. In this context, a country like Pakistan offers an excellent opportunity to study and assess the unique barriers these women must overcome to attain leadership positions in academic medicine and dentistry.

Pakistan is a former British colony and a Muslim-majority country in South Asia with over 241 million population, of whom 48.54% are women [16]. The country’s strictly conservative and patriarchal society strongly influences culture, traditions, and gender dynamics [17] thus significantly reducing women’s opportunities for career choice and progression [18]. The overall job market in Pakistan is male-dominated, and only certain professions, such as teaching and medical practice, are deemed more suitable for women in Pakistan. Women constitute nearly 70% of the students in Pakistani medical schools, but only 50% register with PMDC, and even fewer continue medical practice as a career [19].

There exists a critical gap in our understanding of the gender-based differences in the extent and experience of leaders from a non-Western perspective. In the current study, we investigated the gender-based distribution of academic leaders in medical and dental colleges in Khyber Pakhtunkhwa (KP), Pakistan and explore the experiences of men and women occupying leadership positions in these colleges. The study results would help design gender-based leadership development strategies and promote gender equity in academic institutes across Pakistan.

## Reflexivity

The individuals who conceptualized and designed this study (MS & BJ) hold leadership positions themselves. We are cognizant of both the author’s positions and to avoid our personal biases reflect in the study findings, we were constantly engaged in reflexive practices [20]. For example, the author (MS) wrote personal memos during the data collection, coding, and interpretations. Each transcript was thoroughly discussed, and codes were shared between the researchers. Moreover, before the data analysis started, the coding team set aside and bracketed their personal assumptions to further improve trustworthiness of the study findings.

## Methods

Based on the study objectives, a mixed-method sequential study design [21] including (a) survey of the gender-based distribution of academic leadership positions in medical and dental colleges (b) qualitative interviews with selected individuals occupying leadership positions to explore gender-based differences in their experiences were employed. Integration occurred during development of the interview protocol and results description of both phases to gain an in-depth insight into the extent and experiences of gender based distribution in academic medicine and dentistry. The ethics approval of the study was granted by the institutional research board of Khyber Medical University (Ref No: 1–11/IHPER/MHPE/KMU/23–62) and the study design followed GRAMMS (Good Reporting of a Mixed Methods Study) guidelines.

### Study setting and sampling

The sampling frame for the quantitative survey was all the medical and dental colleges of KP, both public sector and private that have been recognized by the Pakistan Medical & Dental Council. At the time of data collection (June – October 2023), there were 30 colleges in KP offering undergraduate programs in medicine (MBBS) and dentistry (BDS). Information about the gender and rank of those working in academic leadership positions were collected from the official websites of the institutes, followed by confirmation of the organogram with the HR administration of the respective college. Only two private institutes, including a dental college declined to provide the required details and were excluded from the study. Academic leaders were the individuals occupying the position of dean, director or principal, head of the department or chairs. Academic leaders in medical and dental colleges in Pakistan may be divided into three leadership tiers based on roles and responsibilities. The top level includes the dean, directors, or principals responsible for looking after the overall organizational management, including teaching, research, and administration. The mid-level academic leaders take responsibility for a specific educational/training program and include the head of the departments or chairman/women. The line-level leadership is responsible for teaching within the program, including teachers, course coordinators, etc.

For the qualitative arm of the study, we could only get email IDs of 78 academic leaders connected to the undergraduate medical and dental programs at the top and mid-level. The inclusion criteria were persons of any age and gender who were serving in a leadership position for at least one year in a medical and dental college in KP. Those serving in a leadership position on additional/ad-hoc/acting charge who did not meet the required qualifications and experience were excluded from the study. Potential participants sent an email describing the study and an invitation to participate. Only 21 responded positively to our email and they were all included in our final sample. Written informed consent was obtained from all those participants who agreed to participate in the study. Data saturation was observed after 7 interviews [22], but we continue our data collection to ensure equal representation of both genders (5 male, 5 female). A thank you email was sent to the remaining prospective participants informing them that they would not be interviewed as the data saturation has been reached.

### Data collection

For quantitative data collection, college name, status (public or private), and Rank and gender of those working in leadership positions were taken from website and entered Microsoft Excel version 2013. Qualitative data was collected through semi-structured interview sessions conducted by a single researcher (MS) face-to-face or online using the Microsoft ^®^ Team platform depending on the participant choice. Based on the available published literature, a 10-item interview guide was developed and piloted on two participants who were not part of the original study (Supplementary file 1). Information on age, gender, qualification, experience, and current role were also collected. Face-to-face interviews were audio recorded using the record function of a mobile phone device (iPhone 13, Apple inc USA). In the case of the online interview, the recording was conducted using the record option available in the meeting link. To capture the real essence of the experiences, the participants were free to respond in English, Urdu (the national language of Pakistan), and Pashto, most of the interviewees’ mother tongue or first language. Data was securely recorded, translated, and transcribed verbatim.

### Data analysis

**Fig. 1:**
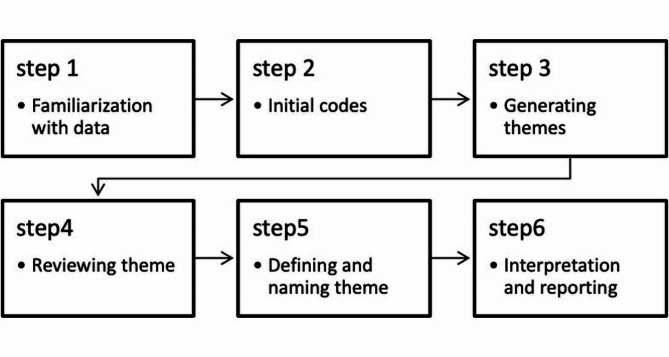
Braun and Clarke thematic analysis steps

The survey data was analyzed using Microsoft Excel 2013 and presented as frequency and percentages. The female-to-male ratio was calculated across different institutes, programs, academic ranks, and departments. Qualitative data was transcribed, and thematic analysis was done using ATLAS Ti Version 8.4.5. The Braun and Clarke framework [21] was used for thematic analysis (Fig. 1). The analysis process involved a systematic approach to coding and interpretation by two researchers (MS & BM). The coding list was further discussed with supervisors (BJ & UM) for improved quality.

## Results

### Quantitative analysis

**Fig. 2:**
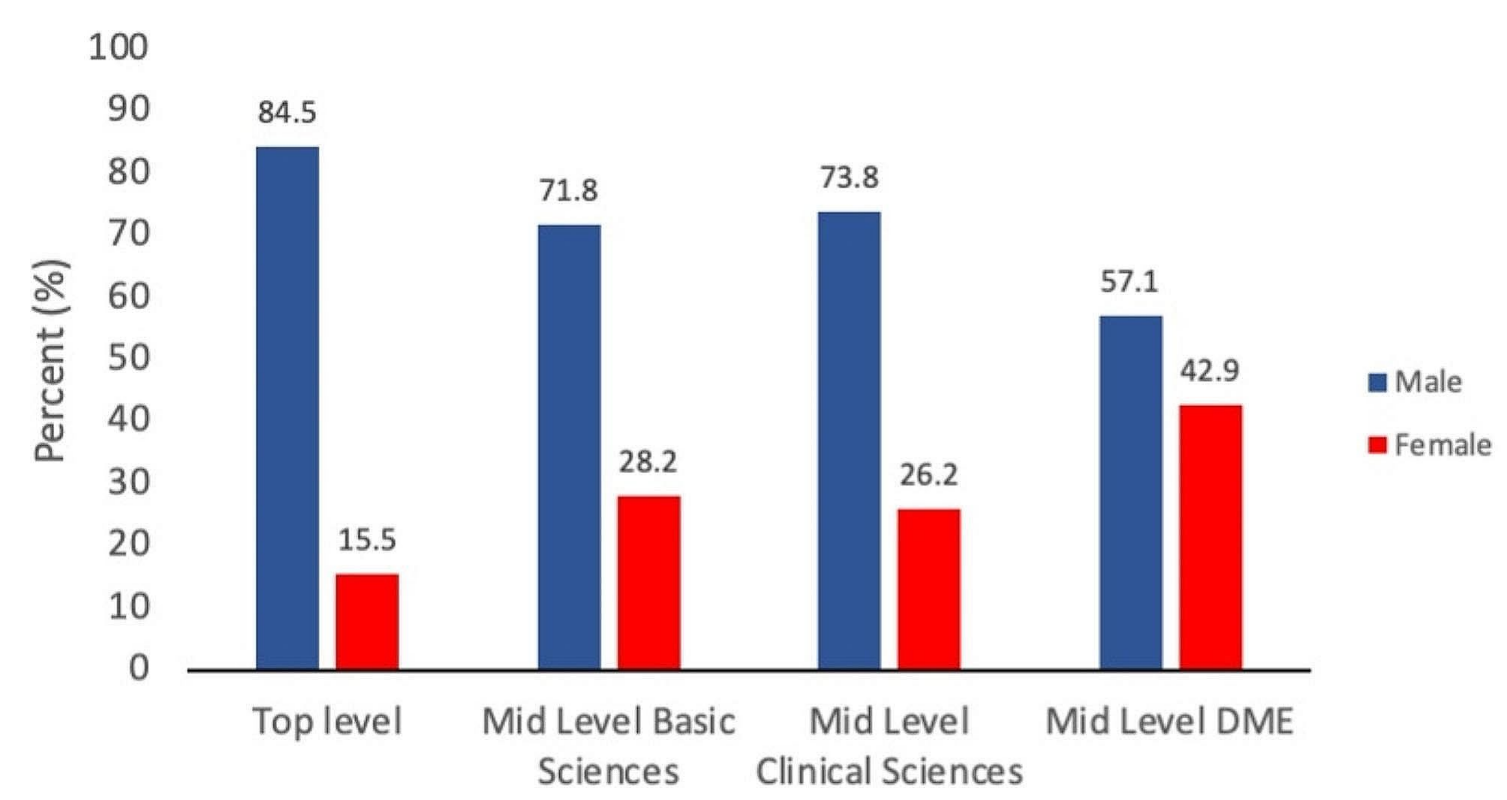
Bar graph showing the gender-based distribution of academic leaders in KP, Pakistan’s medical and dental college

In total, 18 medical and 10 dental colleges including a total of 497 (312 in medicine and 185 in dentistry) academic leaders were surveyed. The overall gender-based distribution of academic leaders is presented in supplementary Tables 1 & 2 and summarized in Fig. 2. Overall, women remain under-represented at all leadership tiers. Gender-based differences were highest at top leadership positions, including principal/deans/directors, where women occupy only 15.5% (*n* = 9) of the total leadership positions. However, women are relatively well represented in mid-level leadership positions. The mid-level leadership positions are further categorized into three groups (basic sciences, clinical sciences, and medical/dental education departments). Across the three categories at the mid-level, the leadership gap tends to be narrower in the medical/dental education department, where more than 40% (*n* = 12) of the heads of the departments were females.

**Fig. 3:**
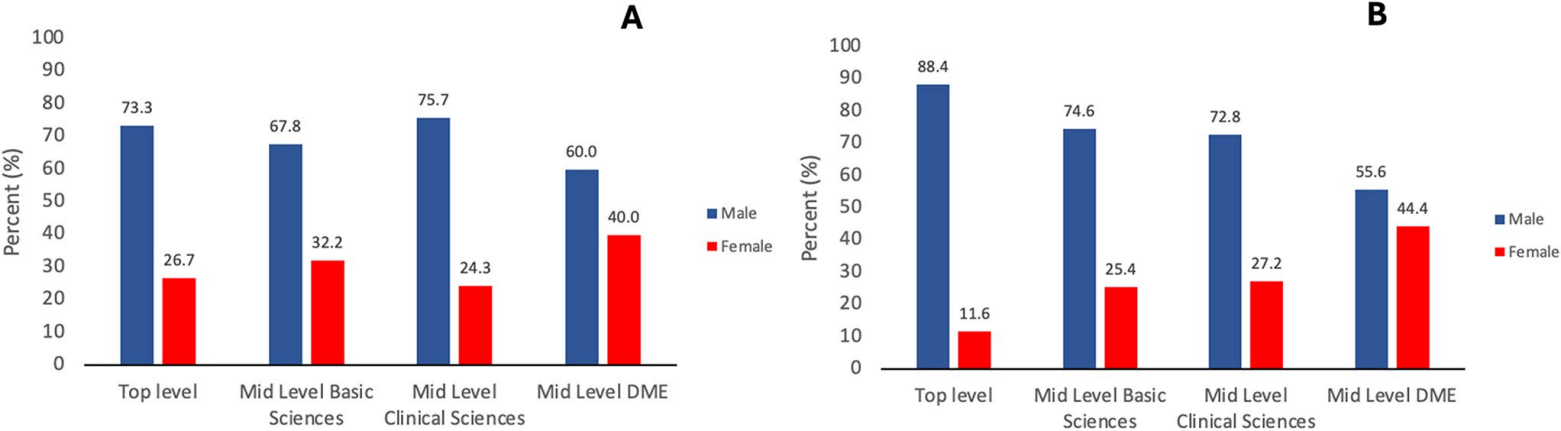
Bar graph representing gender-based distribution at different leadership tiers in (**A**) Dental colleges & (**B**) Medical colleges

We next assessed gender-based differences in academic leadership positions in medical and dental colleges (Fig. 3A & B). At top-level management in dental colleges (Fig. 3A), the percentage of women leaders is almost twice (26.7%; *n* = 4) that of women leaders in medical colleges (11.6%; *n* = 5) (Fig. 3B).

**Fig. 4:**
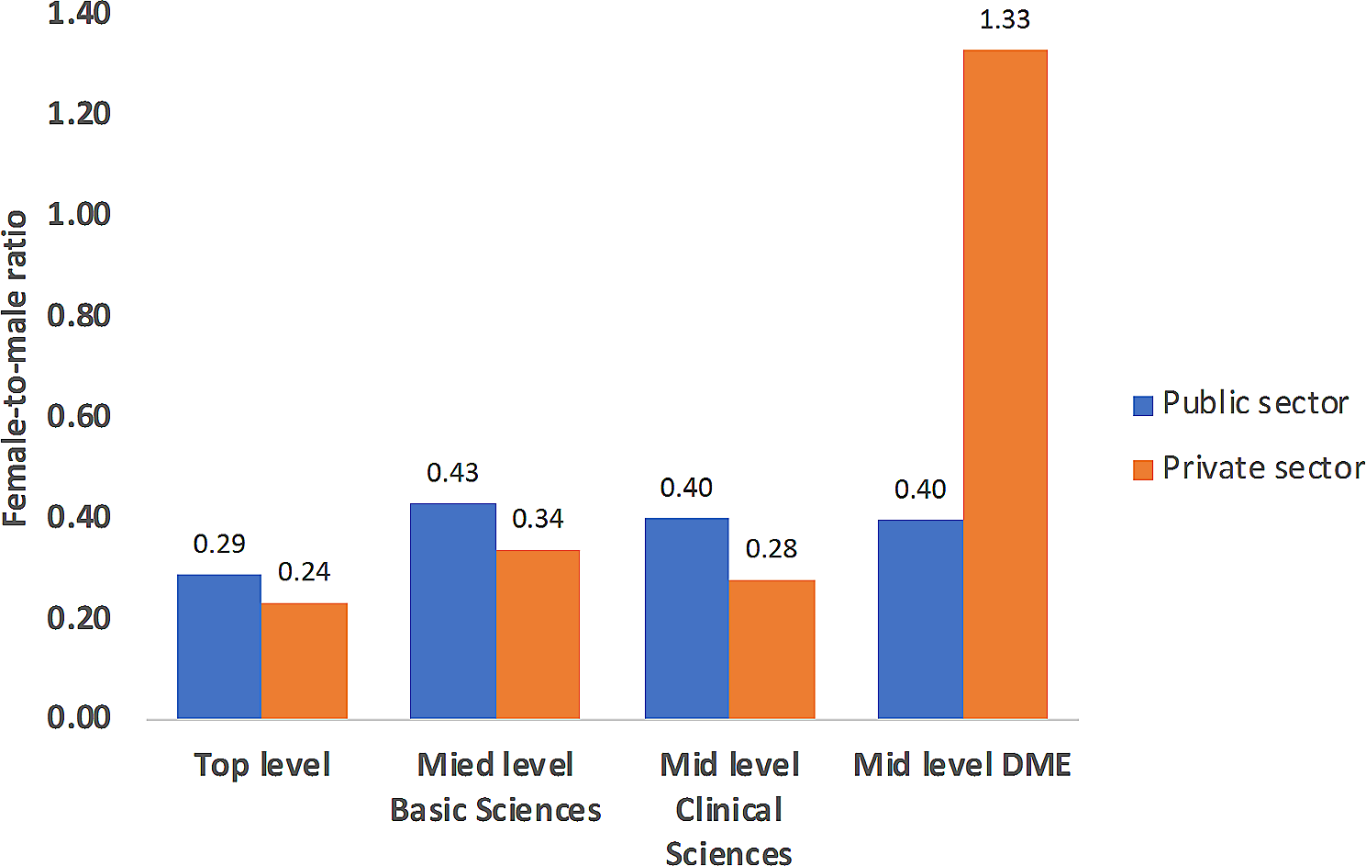
Bar graph representing female-to-male ratio in public and private sector medical and dental college

Gender-based disparities were also observed at the level of the department as well as whether the institute is private or public sector (Fig. 4 & supplementary Tables 1&2). In general, except for medical education, the female-to-male ratio at leadership position was higher in public sector medical (average 0.44; range 0.06–1.71) and dental colleges (average 0.67; range 0.1–1.6) than private sector medical (average 0.36; range 0.14–0.67) and dental (average 0.32; range 0.1–0.5) colleges in the KP province (Fig. 4). Similarly, in certain clinical fields such as obstetrics gynaecology, and prosthodontics, leadership positions were entirely occupied by only one gender.

### Qualitative analysis

Demographic characteristics of the participants are presented in Table 1. Wide variations exist among the participants in terms of age, experience, academic credentials and number of years in a leadership position. The mean age of the participants was 45.4 ± 5.2 years and average experience was 16.4 ± 5.3 years.

**Table 1:** Demographic characteristics of the study participants

Participant ID	Gender	Current institution	Age (years)	Qualification	Experience (Years)	Current role/Position	Years as leader
ID − 1	Male	Public	43	MBBS, MPhil, MPH	17	HOD	6
ID − 2	Female	Public	54	MBSS, FCPS, MHPE	23	Principal	10
ID − 3	Male	Public	47	BDS, FCPS, CHPE	20	Principal	4
ID − 4	Female	Private	49	MBBS, MHPE	13	Director	10
ID − 5	Female	Private	36	PhD	5	Director	2
ID − 6	Female	Public	49	BDS, FCPS, CHPE	18	HOD	2
ID − 7	Male	Public	42	BDS, PhD	12	Director	5
ID − 8	Female	Public	50	MBBS, FCPS, PhD	21	Director	10
ID − 9	Male	Public	42	MBBS, PhD	15	Director	5
ID − 10	Male	Private	42	MBBS, MCPS, FCPS, MSN, PhD, CHPE	20	HOD	10

## Qualitative findings

The experiences of the participants can be described under four main themes and eleven subthemes as presented in Table 2.

**Table 2:** Themes and sub-themes of qualitative analysis

Theme	Sub Theme
Leadership attributes	- Guiding leaders of tomorrow - Flexibility and adaptability - Teamwork and unity
Journey	- Making use of opportunities - Gender discrimination - Benefits of leadership
Challenges	- Work life balance - Family Issues - Overcoming Challenges
Support	- Role of mentor - Building resilience

### Theme 1: Leadership attributes

The participants identified various attributes that are important for a leader. Both male and female participants expressed that a leader is internally motivated to substantially impact the lives of those around them. A vast majority of the participants agreed that they possessed a clear and strong desire to bring about positive transformations in the lives of others through their leadership but also to witness and experience the visible results of their efforts personally.

*If you are a leader*,* you are required to influence people and guide them properly****(ID-1 Male)***

Another leadership attribute identified by participants was good teamwork.

*…One of the leadership qualities is working with your colleagues and subordinates as a team. And when you take them into confidence*,* they support and encourage you and facilitate you in every way…****(ID7- Male)***

Positive mindset, flexibility, diplomacy, and self-awareness were also cited as essential traits for leaders.

*As a leader*,* you are required to know about yourself.…****(ID-1- Female)***

*When you are in a team. Anyone can come up with a good idea*,* an even better idea than yours. You must have the capacity to Embrace that****(ID-10- Male)***

### Theme 2: Leadership journey

The participants in the study shared their views on leadership, stating that it involves multiple responsibilities and requires both education and practical experience. The path to leadership was seen as challenging, but male and female participants had different perspectives on how they achieved it. Female leaders were found to actively seek opportunities, while leadership roles were more readily offered to male participants.

*The transition into leadership roles as a male has been easy****(ID9-Male)***

Female leaders also struggle to balance their domestic responsibilities with their leadership roles, which was not the case for male leaders.

….*I had to do all the house chores myself. The cleaning*,* the cooking*,* the washing*,* the daily activities*,* and then return home to do more work with the kids.****(ID5-Female)***

Both male and female participants acknowledged the existence of gender discrimination in leadership roles.

*When we apply for projects to get funds*,* we are unable to get a percentage like the men.****(ID2-Female)***

*The gender disparity does come into play*,* especially when you’re working as a head of an institute where all the men are working with you*,* even if no one says anything*,* the default thinking is maybe she’s a woman and that’s why she’s saying this****(ID5-Female)***

Participants also highlighted various advantages of leadership in their career growth, including training, financial stability, and autonomy.

…but then they got some advantages to their financially stable, they are socially responsible, you have a strong social background… ***(ID4- Male)***

### Theme 3: challenges

Both male and female participants highlighted the underrepresentation of women in leadership positions.

*Yes*,* because our society is male-dominated. In my own opinion if there was a female in my place*,* she would have surrendered and said that she would have forgiven that….****(ID6- Male)***

Moreover, female participants faced discrimination while pursuing leadership positions. They were able to overcome these challenges by relinquishing some degree of control and seeking assistance from other individuals.

*When I was going for my PhD*,* in my interview*,* they said*,* oh*,* you’re a woman. OK*,* you’re married. Ohh. You have kids. What are you going to do with your kids…****(ID8-Female)***

*….However*,* within the limited end available resources*,* I made efforts with the support from the institute and high-ups****(ID7- Female)***

The challenge of work-family balance was identified by both male and female participants. However the male participants reported that their families were taken care of by their spouses, which allowed them to focus on their work but the female participants had to balance multiple responsibilities.

*My wife is a housewife*,* so that she can give the family time. And my parents are also looked after by my wife. So*,* if financially I’m supporting them*,* the emotional support is always there*,* and it doesn’t make any difference if I am busy****(ID1-Male)***

*…. Yes*,* a terrible effect. It has badly affected my health. My family has excluded me from their social activities and their social circle…****(ID2-Female)***

### Theme 4: support

Mentoring was found to be vital to leaders for their personal growth and advancement as they provided focused guidance and support to aspiring leaders, helping them reach their fullest potential. It was interesting to note that most female participants acknowledged the mentoring role of their father during their leadership journey.

*Mentor for me as a person is my father. He retired as the Chief Secretary of the province. He was a very senior bureaucrat*,* a very well-read person*,* and a scholar…****(ID5- Female)***

Both male and female participants stressed the importance of developing support systems to build resilience. These include the role of institution, family, administration, and collaborative work environment. Interestingly most female participants highlighted that support from female colleagues is necessary to sustain leadership roles.

*A leader cannot become a leader in a vacuum. Your bosses*,* your followers*,* your teammates*,* and your colleagues all have to support a leader to become the leader and there has to be a network of support available in terms of learning opportunities in terms of funding opportunities in terms of Carrying out the vision of the leader****(ID-8 Male)***

*My husband has supported me a lot…****(ID-3 Female)***

*Of Course*,* we don’t get to wherever we are until we have somebody as support*,* especially in academics.****(ID2- Female)***

*Yes*,* and not only that*,* they (female colleagues) give me support at my workplace. We give support to each other…****(ID-5- Female)***

## Discussion

Gender equity and diversity in leadership are crucially important to design and implement policies that ensure a safe learning environment for the students and produce a critical mass of healthcare professionals trained in catering to the needs of a diverse patient population. The integration of results from the quantitative and qualitative arms of the present study suggests that gender- based discrepancies in the extent and experience of academic leader exists in Pakistan. The quantitative analysis showed that women are significantly under-represented especially in the senior level leadership positions where women occupy only 15.5% of the positions. These results follow the same trends as observed in neighbouring South Asian countries such as Bangladesh and India, where women occupy 16% and 18% of the top leadership positions in the healthcare [23, 24]. These findings were further confirmed during the qualitative study reporting gender-based inequalities at both faculty and administration, thus confirming the alarming gender gaps in leadership positions in higher education that not only exist in Pakistan [25] but also in developed countries like the USA, Canada, and UK [15]. The qualitative study also revealed the possible reasons behind the observed gender-based inequalities. For example, the subtheme *“Gender discrimination”* was acknowledge by both male and female participants. The findings that opportunities and sponsorship are more readily available to males than females have also been reflected in the literature previously [26]. Compared to males, it is less likely that a female at a junior rank is recognized for her leadership potential and actively helped (sponsored) in the promotion to a higher rank by a senior who has the power and influence to do so [27]. Another emerging theme was the role of *“mentors”* in the leadership journey. The male participants reported their colleagues, teachers, and clinical supervisors as their mentors, a finding consistent with the previously published literature [28, 29]. However, the female participants frequently reported the role of their father in their academic success and leadership journey. Since Pakistan is a patriarchal society with traditional gender roles in place, this finding is not unsurprising. In the context of gender, the father always plays a major role in helping, guiding, and supporting daughters in pursuit of education, professional excellence or leadership journey [17] especially when they are not married.

Most of the study participant started their leadership journey at the entry-level positions. These findings are in concordance with the study by Tagoe and colleagues [30] who indicate that most of the leadership experiences especially in the case of women leaders, can be gained while working in the middle management positions. The presence of women in mid-level leadership positions and their preference for academic medicine [10] means that gender disparities in high-level positions will gradually be reduced in future.

Our study report comparatively more women in mid-level (Fig. 1) than in the senior level leadership positions. During the qualitative interviews, the participants especially the women leaders revealed that they are struggling to maintain a work-life balance. Long working hours (sometimes exceeding 80 h per week) along with domestic responsibilities such as taking care of the children and family [31] force women to remain in middle rather than senior leadership positions [32, 33]. These issues also force a lot of lady doctors in Pakistan to quit their job [18].

The uneven gender-based distribution of academic leaders was also observed between private and public sector institutes as well as individual departments within the same institute. In general, except for medical education, the female-to-male ratio was higher in public sector medical and dental colleges of the KP province. These findings are similar to recently published reports from Sweden and the USA, indicating a narrower gender gap in the public than private sector [34, 35].

Similarly, in certain clinical fields such as obstetrics & gynecology, and prosthodontics, leadership positions were entirely occupied by only one gender. This gender polarization is not surprising. Due to cultural norms and traditions, female patients mostly prefer women doctors for obstetrics and gynae services [36], thereby making it the field of choice for women doctors in Pakistan [37, 38]. On the contrary, the number of women leaders in surgery and allied were disproportionally low in the current study. Historically, surgery is considered a masculine field, and women are scarce in leadership positions across the globe, even in developed countries like the USA [39].

## Conclusions

This study found that gender disparities exist at leadership positions as well as experiences in medical and dental colleges of KP Pakistan. The women leaders frequently reported gender-based inequality experiences and challenges in maintaining a decent work-life balance due to multiple domestic responsibilities. Although the majority of the top-level leadership positions are occupied by men, the relatively high representation of women at the mid-level indicates that the gender-based disparities in leadership could be more balanced in the future. These finding are critical and can have important implications for government, organizations and policy makers in term of enacting laws, propose regulations and establish support mechanism that help current and aspiring leaders in their leadership journey.

### Strengths and limitations

Our study provides a multi-faceted perspective on the extent and experiences of gender-based disparities in medical and dental institutes in Pakistan. The inclusion of all medical and dental institutes increased the generalizability of the study findings while the more than 93% response rate of the survey reduced the selection bias thus increasing validity. Moreover, the qualitative arm of the study provided deep insights into the experiences of academic leaders in a developing and strictly patriarchal society. However, our study has some limitations. The study findings cannot be generalized across Pakistan due to widely prevalent differences across different ethnicities, cultures, and socioeconomic background. Another limitation of the study that should be considered is that the survey information was collected based on department-wise availability of leadership roles. However, the subdivisions in some academic departments for example Medicine & Allied were not included in our study and therefore, variations are expected in the exact female-to-male ratio in the medical and dental colleges.

### Electronic supplementary material

Below is the link to the electronic supplementary material.

Supplementary Material 1

Supplementary Material 2

Supplementary Material 3

## Data Availability

This published article and its supplementary information files include all data generated or analyzed during this study.
