# Supplementary material for: Gender-based differences in the representation and experiences of academic leaders in medicine and dentistry: a mixed method study from Pakistan
Source: BMC Med Educ. 2024 Aug 16;24:885. doi: 10.1186/s12909-024-05811-6 (PMC11330139; doi:10.1186/s12909-024-05811-6)
Supplement: Supplementary file 3 — Supplementary Material 3 [file 12909_2024_5811_MOESM3_ESM.docx]

Supplementary File 1: Semi-structured interview guide for academic leaders in medical and dental colleges of Khyber Pakhtunkhwa, Pakistan

| Themes | Guiding questions |
| --- | --- |
| Opening question(s)  Introduction to leadership | **1.** How you define leadership based on your own experience. |
| Day to day life/Living with busyness/Daily activities in leadership role | **2.** Tell me about your role and responsibilities as leader/head of the institute.   - Teaching, administration, research? - What do you like/enjoy most about your job? - What you don’t like/enjoy least in your job? |
| Journey to leadership position/Leadership pathway | **3.** Tell me about your journey starting from graduation till now.  If needed, follow up with   - Education and professional development journey (degrees, courses etc). - Work experience starting from first job till now. - What was the process that got you into current leadership role? - What experiences were more important? Education or on-job training? Else? |
| Challenges and opportunities | **4.** From your experience, what opportunities/support were available to you   - At the level of institute - At family and society level   **5.** From your experience, what barriers/challenges did you experience (institute, society level) and how did you overcome them?  **6.** Has your gender influenced or affected your career? If yes, how? Positive or negative? |
| Mentorship & support | **7:** Do you have a mentor who has supported or guided you in achieving the leadership role?   - What advice have they given you?   **8:** How well are you supported by your institute in pursuing your leadership activities? |
| Workplace domain | **9.** What do you think about your working environment at the institute? Positive or negative or mix  If needed, follow up with   - Flexibility or work-life balance - Financial gain/salary package - Support from team members - Career development opportunities - Diversity/gender mix in your organization? - How does it feel to be a [woman/ male] leader in your institution? |
| Family domain | **10.** Tell me about your home life? Family, kids etc.   - Daily routine activities/responsibilities at home - Do your leadership responsibilities at your work place affect your home life? How? Positive, negative or both? Explain please |
| Closing statement | Thank you for your time and effort to make this interview a success. I will send a  copy of the transcription to you for the accuracy of all information given. I will also send you a completed copy of the research study once it is finalized. |
